# Survival analysis of factors affecting the timing of COVID-19 non-pharmaceutical interventions by U.S. universities

**DOI:** 10.1186/s12889-021-12035-6

**Published:** 2021-11-02

**Authors:** Kevin E. Cevasco, Amira A. Roess, Hayley M. North, Sheryne A. Zeitoun, Rachel N. Wofford, Graham A. Matulis, Abigail F. Gregory, Maha H. Hassan, Aya D. Abdo, Michael E. von Fricken

**Affiliations:** 1 Department of Global and Community Health, George Mason University, 4400 University Drive, Fairfax, VA 22030 USA; 2 Department of Biology, College of Science, George Mason University, Fairfax, VA USA

**Keywords:** COVID-19, Non-pharmaceutical interventions, U.S. universities, Pandemic response, Timing

## Abstract

**Background:**

During March of 2020 the Centers for Disease Control and Prevention (CDC) announced non-pharmaceutical intervention (NPI) guidance as the primary mitigation strategy against growing COVID-19 community spread due to the absence of a vaccine or effective treatment at that time. CDC guidance states that NPIs are most effective when instituted in an early, targeted, and layered fashion. NPIs are effective in slowing spread, and measures should be custom-tailored to each population. This study examines factors associated with implementation and timing of NPI interventions across large public and private U.S. universities at the onset of the COVID-19 pandemic.

**Methods:**

NPI decisions of interest include when U.S. universities canceled international travel, shifted to online learning, moved faculty/staff to remote work, limited campus housing, and closed campus for all non-essential personnel. Cox proportional hazard analyses of retrospective data were conducted to assess the time to NPI events. Hazard ratios were calculated for university governance, campus setting, religious affiliation, health infrastructure, faculty diversity, and student demographics. The methods control for variance inflation factors, COVID case prevalence, and time varying covariates of spring break and states’ state of emergency (SOE) orders. This study captures NPI decisions at 575 U.S. universities during spring of 2020 which affected the movement of seven million students and two million employees.

**Results:**

Universities located in districts represented by Democratic party congressional members reported earlier NPI implementation than Republican (Cox proportional hazard ratio (HR) range 0.61–0.80). University religious affiliation was not associated with the timing any of the NPI decisions. Universities with more diverse faculty showed an association with earlier NPI implementation (HR range 0.65–0.76). The existence of university-affiliated health infrastructure was not associated with NPI timing.

**Conclusion:**

University NPI implementation was largely driven by local COVID-19 epidemiology, culture and political concerns. The timing of university NPI decisions varied by regional politics, faculty demographics, university governance, campus setting, and foreign student prevalence adjusting for COVID-19 state case prevalence and spring break timing. Religious affiliation and presence of university health infrastructure were not associated with timing.

**Supplementary Information:**

The online version contains supplementary material available at 10.1186/s12889-021-12035-6.

## Background

At the onset of the COVID-19 pandemic, national and international health agencies and governments relied solely on non-pharmaceutical interventions (NPIs) due to the absence of effective therapeutics and vaccines at the time, and continued to do so given the rapid emergence of several variants to which current therapeutics and vaccines may not be effective against [1]. Key strategies, such as restrictions on movement and large gatherings were deployed broadly to mitigate COVID-19 community spread [2]. Since March 2020, state and local governments have implemented various forms of NPI guidance, including cancelation of sponsored travel, stay-at-home (SAH) orders, and school closures. During March 1–May 312,020, 42 states and territories issued mandatory stay-at-home orders, affecting 2355 (73%) of 3233 U.S. counties [3].

During the initial stages of this pandemic, university communities across the U.S. were also faced with the challenge of interpreting and implementing NPI guidance from local, state, and national policymakers and agencies. University leadership had to determine whether, and when, to 1) move to online learning, 2) limit campus housing, 3) cancel international travel, 4) close campus for all non-essential personnel, and 5) shift faculty/staff to remote work. The Centers for Disease Control and Prevention (CDC) published an interim pandemic mitigation framework in 2007 with updates in 2017 [4–6]. The guidance asserts that these NPIs are most effective when instituted in an early, targeted, and layered fashion [4]. NPI adoption and decision making was often made at the university level, where leadership balanced financial ramifications of NPI implementation and the health and safety of employees and students. CDC guidance encourages universities to regularly share their pandemic response decisions with community partners and stakeholders [5].

Influences on university NPI adoption timing have not been well studied, and research suggests a link between community leadership and NPI adherence [7]. Studies on Ebola epidemics globally have demonstrated that NPI compliance benefits from working with local community leaders in the decision making and implementation process [7]. The heterogeneity of infection by socio-demographic stratifications, and intergenerational contacts across different settings suggests that one-fits-all NPIs strategies are not optimal. Measures should be custom-tailored to the specific context of each population [8]. In the case of COVID-19, decisions at 575 universities with 5000 or more students affected the social distancing of 2 M employees and 7 M students [9]. University leadership decision-making is influenced by a broad network of stakeholders, including state administrators, boards of visitors, and legal teams where university leadership buffers their organizations against budget cuts, aggressive oversight, and liability [10].

This study aim is to determine cultural and socio-demographic factors affecting the timing of NPI decisions at U.S. universities at the onset of the COVID-19 pandemic. A secondary objective is to provide detailed results that allow policy makers and health officials to compare influence of state level public health orders, COVID-19 prevalence, and timing of spring break.

## Methods

### Study design

This study assesses factors associated with timing of NPIs at U.S. universities using an original dataset of university COVID-19 decisions [9]. The COVID-19 dataset of non-pharmaceutical interventions across U.S. universities, March 2020 contains data on key NPI decisions and demographics of universities. These universities were of interest because they are often critical parts of society playing key roles in education, innovation and economic growth in their communities. They are complex entities that operate study abroad programs, conduct international research, admit a high volume of international students, and have a larger on-campus student population. Community colleges, online-only institutions, and vocational programs were excluded because they often lack on-campus housing, may not need to transition on-line, and less frequently offer study abroad programs.

This study considers universities from all 50 states and the District of Columbia, representing a variety of geographic settings, institutional characteristics, and subpopulations. Data were collected directly from university COVID-19 webpages, university social media accounts, and other sources to extract precise datum on decision timelines for NPI interventions, as previously described [6, 9]. Most decisions were communicated publicly in the form of official policy announcements by university leadership labeled with dates the announcements were made. Publicly available sites captured the chronology of official announcements typically originating from the university President or Provost offices and includes university announcements.

Key public health announcement and guidance dates: Department of Education Guidance for interruptions of study related to Coronavirus disease (COVID-19) released on March 5th. CDC Guidance for Institutions of Higher Education with Students Participating in International Travel or Study Abroad Program released on March 9th. CDC Interim Guidance for Administrators of US Institutions of Higher Education released on March 18th. On March 11th the WHO issued the Pandemic declaration. Additionally, on March 13th, the White House declared a national emergency.

### Variables

Outcome variables are based on NPI decisions from published guidance by the Department of Education, CDC, and other national and international agencies. The five outcome variables of interest are: 1) “Move online” which indicates that the university announced all classes will be conducted in an online/distance learning format, whether for a few weeks or the rest of the semester; 2) “Discourage campus housing” indicates that the university encouraged students to leave or not come back to campus housing; 3) “Cancel travel” indicates that the university decided to cancel/suspend/prohibit all university-sponsored travel; 4) “Close campus” indicates that the university limited campus access to essential/mandatory personnel only; 5) “Remote work” indicates that remote work/telecommuting was the default option for staff/faculty.

Explanatory variables are based on themes from a systematic review on public perceptions of NPI adoption [11]. A systematic review of NPIs showed that the public’s individual adoption of respiratory infection-related NPIs varies based on five key themes: 1) personal and cultural beliefs about infection transmission, 2) diagnostic uncertainty with emerging respiratory infections, 3) perceived vulnerability to infection, 4) anxiety about emerging respiratory infections and 5) communication about emerging respiratory infections [11].

Explanatory variables include political party affiliation of the local Congressional House of Representatives member, political party affiliation of the Governor, university religious affiliation, racial diversity of faculty, racial diversity of students, prevalence of foreign students, and private versus public governance. Diversity variables are calculated as proportion of non-white faculty and students. Health infrastructure was measured through the presence of at least one of the following at the university: school of public health, medical school, university affiliated hospital, or Master of Public Health (MPH) program. Campus environment IPEDS categories include city, suburban, town and rural settings. University size is based on amounts of enrolled students. A university’s state case prevalence is included because CDC guidance on implementing NPIs calls for an assessment level of community spread [12].

### Statistical analysis

Descriptive statistics show frequency and mean of the NPI outcome variables and explanatory variables. Survival analyses were conducted to assess the time to NPI decision event for each of the five outcomes of interest against explanatory variables. Binary explanatory variables were created to assess the hazard ratio (HR) for the universities included in this study. Comparisons include public versus private institutions, religious versus non-religious affiliation, existence of a university health infrastructure, political party affiliation of Governor, and political party affiliation of Congressional House of Representatives member. Several variables were split at the mean to create binary variables of universities showing more/less diverse faculty, more/less racially diverse students, and fewer/more foreign students. Campuses in city/suburban settings are compared to town/rural settings. Universities were split into medium (> 5000 and < 20,000) and large (> 20,000) count of enrolled students.

Hazard ratio measures were used to determine how often an NPI decision happened in one group compared to how often it happened in another group over time. Cox regression survival analysis was performed comparing universities that implemented an NPI to those that did not implement an NPI. University decisions were recorded on discrete days, with the Efron option used to address common same day decisions [13].

The impact of the prevalence of COVID-19 cases on university decisions was measured by the number of positive cases in the state on the date when an NPI decision was announced, with universities assigned to a state case prevalence quartile for each NPI outcome (Q1-lowest, Q4-highest cases). Statistics and survival analysis were conducted using Stata version 16 (StataCorp. College Station, TX).

### Models

Five models assessed the associations between NPI outcomes and explanatory variables. A log rank test determined if the proportional hazard assumption was met for all variables in each model, which accounts for changes in decision trends based on published public health guidance during the study period. Explanatory variables that did not meet the proportional hazard assumption were removed from models. State COVID case prevalence at time of each NPI decision is represented in by placing universities into state based quartiles. Quartiles enable a state to state severity comparison given the exponential case count growth. Alternative models to adjust for the impact of spring break and state-level state of emergency (SOE) and state level safer at home (SAH) orders were assessed to determine if they are time varying covariates. The dates for university spring breaks were normalized to the first Monday of each break. A variance inflation factor checked for collinearity of coefficients in the Cox models. Alternate Cox models were then stratified to assess potential collinearity of covariates.

It is not known if the missing NPI announcement data reflects a university decision made after the observation period or a decision not to implement an NPI policy. Missing data for each NPI outcome variable was assumed out of the study time range and universities with missing data were truncated out of each Cox model. To assess potential bias in this missing data approach a supplementary table (Table S1) is provided where missing dates were set to the end of the observed study time range (March 31, 2020).

## Results

### Descriptive statistics

A total of 575 universities were included in the data set representing 7,044,968 students and 2,050,411 employees across 50 states and District of Columbia. The study captured 100% of university decisions to move online, and between 89 and 95% of the other four NPI decisions variables, with specific details on counts found in Table 1. All universities implemented NPIs, where 75% of universities publicly announced implementation all five NPI measures, 93% implemented four mitigations, and 98% implemented at least three. Most universities have less than 20 k enrolled students (423, 73.6%), and are in districts represented by Democratic Congressional House of Representatives (320, 55.7%). Additionally, the majority of included universities were public (409, 71.1%) and not religiously-affiliated (503, 87.5%). City and suburban based universities (482, 83.8%) were more common than town and rural universities (93, 16.2%), and a majority of included universities did not have health-related programs, hospitals, or medical school infrastructure (368, 64%).

**Table 1:** Descriptive statistics of universities that implemented each of the NPIs by explanatory variable categories

Number of Universities, *N* = 575	Non-pharmaceutical interventions to restrict movement (n (%))				
Explanatory variables	Move online	Campus housing	Cancel travel	Campus closed	Remote work
Private	166 (100.0%)	153 (92.2%)	151 (93.2%)	144 (91.1%)	151 (94.4%)
Public	409 (100.0%)	355 (86.8%)	388 (97.2%)	366 (91.3%)	396 (97.5%)
< 20 k students enrolled	423 (100.0%)	367 (86.8%)	390 (94.9%)	371 (90.7%)	399 (96.1%)
> 20 k students enrolled	152 (100.0%)	141 (92.8%)	149 (99.3%)	139 (92.7%)	148 (98.0%)
House Democrat	320 (100.0%)	280 (87.5%)	298 (95.2%)	295 (93.7%)	312 (98.1%)
House Republican	247 (100.0%)	221 (89.5%)	233 (97.1%)	208 (87.8%)	227 (94.6%)
Governor Democrat	321 (100.0%)	282 (87.9%)	302 (95.6%)	294 (93.6%)	309 (97.2%)
Governor Republican	254 (100.0%)	226 (89.0%)	237 (96.7%)	216 (88.2%)	238 (96.0%)
More Diverse faculty	235 (100.0%)	197 (83.8%)	216 (94.7%)	213 (93.8%)	227 (98.7%)
Less Diverse Faculty	340 (100.0%)	311 (91.5%)	323 (97.0%)	297 (89.5%)	320 (95.2%)
More Diverse students	257 (100.0%)	213 (82.9%)	234 (93.2%)	232 (92.1%)	247 (96.5%)
Less Diverse Students	318 (100.0%)	295 (92.8%)	305 (98.4%)	278 (90.6%)	300 (96.8%)
Religious Affiliation	72 (100.0%)	69 (95.8%)	65 (94.2%)	62 (89.9%)	65 (94.2%)
No Religious affiliation	503 (100.0%)	439 (87.3%)	474 (96.3%)	448 (91.4%)	482 (97.0%)
Health Infrastructure	207 (100.0%)	191 (92.3%)	199 (97.1%)	192 (93.7%)	202 (98.5%)
No Health Infrastructure	368 (100.0%)	317 (86.1%)	340 (95.5%)	318 (89.8%)	345 (95.6%)
Fewer Foreign Students	290 (100.0%)	247 (85.2%)	267 (95.0%)	246 (88.2%)	272 (95.8%)
More Foreign Students	285 (100.0%)	261 (91.6%)	272 (97.1%)	264 (94.3%)	275 (97.5%)
City/Suburb	482 (100.0%)	427 (88.6%)	453 (96.0%)	433 (92.1%)	463 (97.3%)
Town/Rural	93 (100.0%)	81 (87.1%)	86 (96.6%)	77 (86.5%)	84 (93.3%)

Within each cell, “n” represents the number of universities that implemented the mitigation indicated by the column heading. The “(%)” represents the percentage that implemented the mitigation relative to the total number of universities in that explanatory variable category. Percentages reflect missing data which causes variation in percentages across columns

Table 2 shows that NPI announcements occurred over several weeks during the spring of 2020. The chronological order of NPI decisions differed between mean announcement date and mean case prevalence. Universities in the first 3 quartiles implemented all five NPIs before the total mean cases, with only Q4 NPI decisions occurring after cases rose above the mean.

**Table 2:** Mean date of NPI announcement with state case prevalence quartile on day of announcement

Case prevalence quartile	Move online	Cancel travel	Campus housing	Close campus	Remote work
Mean date NPI announced | Variance in days					
Q1	Mar-11 | 1	Mar-06 | 3	Mar-11 | 1	Mar-16 | 2	Mar-14 | 2
Q2	Mar-11 | 1	Mar-10 | 2	Mar-12 | 2	Mar-19 | 3	Mar-15 | 2
Q3	Mar-12 | 2	Mar-11 | 2	Mar-15 | 3	Mar-20 | 3	Mar-16 | 3
Q4	Mar-11 | 2	Mar-13 | 4	Mar-17 | 3	Mar-21 | 4	Mar-17 | 4
Total	Mar-11 | 2	Mar-10 | 4	Mar-14 | 3	Mar-19 | 4	Mar-15 | 3
Number of positive cases in state on day of decision | Variance in cases					
Q1	3 | 2	0 | 0	4 | 3	32 | 18	150 | 327
Q2	12 | 4	5 | 2	20 | 5	157 | 63	451 | 1189
Q3	37 | 19	21 | 8	72 | 42	471 | 126	748 | 2048
Q4	339 | 382	800 | 1746	1633 | 4031	3561 | 4322	2853 | 4118
Total	97 | 236	206 | 934	430 | 2122	1042 | 2586	1052 | 2602

### Survival analysis

Table 3 shows the results of a Cox proportional hazard regression with binomial and categorical explanatory variables. The table is arranged by the five NPI models with explanatory variables. Universities with private governance announced earlier moves to online learning than public universities (HR = 0.79, 95% CI 0.64:0.98, *p* = 0.031), Democratic Governors earlier than Republican (HR = 0.80, 95% CI 0.73:0.88, *p* < 0.001), Congressional house districts represented by Democratic members earlier than Republican (HR = 0.64, 95% CI 0.52:0.78, *p* < 0.001), and more diverse faculty earlier than less diverse (HR = 0.76, 95% CI 0.60:0.95, *p* = 0.017). Universities with fewer foreign students moved online later (HR = 1.22, 95% CI 1.02:1.46, *p* = 0.029). Decisions to move online were not positively associated with COVID-19 state case prevalence. The order of chance was non-linear with universities in Q4 (HR = 0.53, 95% CI 0.40:0.69, *p* < 0.001) showing a greater chance than Q3 (HR = 0.39, 95% CI 0.30:0.50, *p* < 0.001).

**Table 3:** Hazard ratios for NPI and explanatory variables

Survival analysis models with explanatory variables	Relative risk (95% confidence interval)	P
*Move Online*		
Private / Public	**0.79 (0.64:0.98)**	**0.031**
Democrat/Republican Governor	**0.80 (0.73:0.88)**	**< 0.001**
Democrat/Republican Congressional House	**0.64 (0.52:0.78)**	**< 0.001**
More / Less Diverse Faculty	**0.76 (0.60:0.95)**	**0.017**
More / Less Diverse Students	1.09 (0.87:1.37)	0.466
No/ Yes Health infrastructure	0.89 (0.72:1.09)	0.257
Fewer/more foreign students	**1.22 (1.02:1.46)**	**0.029**
City-Suburb / Town-Rural	0.92 (0.72:1.18)	0.517
Enrollment < 20 k / > 20 k	1.25 (0.99:1.57)	0.059
State Case Prevalence, Quartile 1	1	
Quartile 2	0.82 (0.64:1.05)	0.124
Quartile 3	**0.39 (0.30:0.50)**	**< 0.001**
Quartile 4	**0.53 (0.40:0.69)**	**< 0.001**
*Campus Housing*		
Private / Public	**0.50 (0.38:0.65)**	**< 0.001**
Religious / Not Religious	1.26 (0.90:1.77)	0.171
Democrat/Republican Congressional House	**0.70 (0.57:0.86)**	**< 0.001**
No/ Yes Health infrastructure	0.99 (0.81:1.19)	0.879
Fewer/more foreign students	0.94 (0.77:1.13)	0.495
City-Suburb / Town-Rural	**0.76 (0.58:1.00)**	**0.048**
State Case Prevalence, Quartile 1	1	
Quartile 2	**0.54 (0.42:0.70)**	**< 0.001**
Quartile 3	**0.22 (0.16:0.29)**	**< 0.001**
Quartile 4	**0.11 (0.08:0.15)**	**< 0.001**
*Cancel Travel*		
Private / Public	0.83 (0.67:1.03)	0.087
Democrat/Republican Congressional House	**0.61 (0.50:0.73)**	**< 0.001**
Fewer/more foreign students	1.00 (0.83:1.20)	0.994
State Case Prevalence, Quartile 1	1	
Quartile 2	**0.41 (0.32:0.53)**	**< 0.001**
Quartile 3	**0.30 (0.23:0.39)**	**< 0.001**
Quartile 4	**0.14 (0.10:0.18)**	**< 0.001**
*Campus Closed*		
Religious / Not Religious	1.37 (1.03:1.82)	^a^0.032
Democrat/Republican Governor	**0.77 (0.70:0.85)**	**< 0.001**
Democrat/Republican Congressional House	**0.67 (0.54:0.83)**	**< 0.001**
More / Less Diverse Faculty	**0.65 (0.53:0.80)**	**< 0.001**
State Case Prevalence, Quartile 1	1	
Quartile 2	**0.32 (0.25:0.42)**	**< 0.001**
Quartile 3	**0.21 (0.16:0.27)**	**< 0.001**
Quartile 4	**0.11 (0.08:0.16)**	**< 0.001**
*Remote Work*		
Democrat/Republican Governor	**0.68 (0.62:0.75)**	**< 0.001**
Democrat/Republican Congressional House	**0.77 (0.62:0.94)**	**0.011**
More / Less Diverse Faculty	**0.69 (0.54:0.88)**	**0.003**
More / Less Diverse Students	0.86 (0.67:1.10)	0.226
No/ Yes Health infrastructure	0.89 (0.72:1.09)	0.262
City-Suburb / Town-Rural	**0.76 (0.58:0.99)**	**0.043**
Enrollment < 20 k / > 20 k	**1.34 (1.07:1.67)**	**0.010**
State Case Prevalence, Quartile 1	1	
Quartile 2	**0.52 (0.40:0.67)**	**< 0.001**
Quartile 3	**0.25 (0.19:0.33)**	**< 0.001**
Quartile 4	**0.16 (0.12:0.21)**	**< 0.001**

Statistically significant results in bold text

^a^Campus closure, Religious / Not Religious was not statistically significant (*p* = 0.087) when adjusting for spring break as a time varying covariate

Several factors were associated with earlier move away from on-campus housing where private universities moved earlier than public (HR = 0.50, 95% CI 0.38:0.65, *p* < 0.001), Congressional house districts represented by Democratic members earlier than Republican (HR = 0.70, 95% CI 0.57:0.86, *p* < 0.001). Universities with campuses in city-suburban settings moved earlier than town-rural (HR = 0.76, 95% CI 0.58:1.00, *p* = 0.048). Decisions to limit campus housing were not positively associated with COVID-19 state case prevalence (Q2 HR = 0.54, 95% CI 0.42:0.70, *p* < 0.001; Q3 HR = 0.22, 95% CI 0.16:0.29, *p* < 0.001; Q4 HR = 0.11, 95% CI 0.08:0.15, *p* < 0.001).

The only factor associated with cancelling travel was Congressional house political party affiliation where districts represented by Democratic members moved earlier than Republican (HR = 0.61, 95% CI 0.50:0.73, *p* < 0.001). Decisions to cancel travel were not positively associated with COVID-19 state case prevalence (Q2 HR = 0.41, 95% CI 0.32:0.53, *p* < 0.001; Q3 HR = 0.30, 95% CI 0.23:0.39, *p* < 0.001; Q4 HR = 0.14, 95% CI 0.10:0.18, *p* < 0.001).

Several factors were associated with earlier campus closure. Universities with Democrat Governors closed earlier than Republican (HR = 0.77, 95% CI 0.70:0.85, *p* < 0.001), Congressional house districts represented by Democratic members closed earlier than Republican (HR = 0.67, 95% CI 0.54:0.83, *p* < 0.001). Universities with more diverse faculty closed earlier than less diverse (HR = 0.65, 95% CI 0.53:0.80, *p* < 0.001). Decisions to close campus were not positively associated with COVID-19 state case prevalence (Q2 HR = 0.32, 95% CI 0.25:0.42, *p* < 0.001; Q3 HR = 0.21, 95% CI 0.16:0.27, *p* < 0.001; Q4 HR = 0.11, 95% CI 0.08:0.16, *p* < 0.001).

Universities in states with Democrat Governors moved employees to remote work earlier than Republican (HR = 0.68, 95% CI 0.62:0.75, *p* < 0.001), Congressional house districts represented by Democratic members moved to remote work earlier than Republican (HR = 0.77, 95% CI 0.62:0.94, *p* = 0.011). Universities with more diverse faculty moved to remote work earlier than less diverse (HR = 0.69, 95% CI 0.54:0.88, *p* = 0.003). Campuses in city-suburban settings moved to remote work earlier than town-rural (HR = 0.76, 95% CI 0.58:0.99, *p* = 0.043); and those with student enrollment less than 20 k were later (HR = 1.34, 95% CI 1.07:1.67, *p* = 0.010). Decisions to move to remote work were not positively associated with COVID-19 state case prevalence (Q2 HR = 0.52, 95% CI 0.40:0.67, *p* < 0.001; Q3 HR = 0.25, 95% CI 0.19:0.33, *p* < 0.001; Q4 HR = 0.16, 95% CI 0.12:0.21, *p* < 0.001).

Alternate models analyzed potential covariates among explanatory variables which stratified the correlated variables but showed no change in statistical significance nor hazard ratios. In the model to move online there is moderate correlation with faculty and student diversity (− 0.54). There was weak correlation with 20 k enrollment and health infrastructure (− 0.45), and Governor’s political party and Q4 prevalence (0.30). For the campus housing model there is a moderate correlation with public and religious universities (− 0.61). No correlation was shown in the cancel travel model. For campus closure there was moderate correlation with faculty diversity and congressional house member party (− 0.38). Remote work showed moderate correlation with faculty and student diversity (−.60) and 20 k enrollment with health infrastructure (− 0.43).

Neither health infrastructure nor student diversity were statistically significant in any of the models. Adjusting for spring break as a time-varying covariant resulted in religious-affiliated university campus closure becoming not statistically significant (*p* = 0.087) but had no other material effect on the results. Introducing state level state of emergency (SOE) and safer at home (SAH) NPIs as time varying covariates the Cox models resulted in neither SOE nor SAH being statistically significant. Supplemental Table (S1) shows results with missing NPI decisions set to occur at the end of the observation period (March 31st, 2020). The supplemental table shows similar results to primary Cox models (Table 3) with only minor variations in ratios and confidence intervals. In Table S1 the urban setting of campus housing was the only variable that became non statistically significant versus Table 3.

## Discussion

Universities generally implemented government guidance using targeted, layered application of multiple partially effective non-pharmaceutical interventions before explosive COVID-19 community spread. CDC guidance recommends universities consider background rates of community infection when making NPI decisions. However, university decision timing was not positively associated with increasing COVID-19 state case prevalence [14]. This may be partly due to early and rapid community spread before public health orders and guidance were issued. Many universities used their autonomy to implement NPIs prior to national and state public health guidance and orders. For example, CDC issued International Travel or Study Abroad Program guidance on March 9th 2020 when many states had outbreaks with over 100 cases (NY, NJ, CA, MI, and WA). Many universities announced travel cancellation with elevated cases in their state.

Universities in Congressional districts with Democratic party representation implemented NPIs earlier. Other studies support the finding that Republican Governors delayed implementing COVID-19 NPIs compared to states with Democratic Governors [15]. Research shows that ideology of a region’s elected legislators predicts the political ideology of a population at the state, congressional district, and state legislative district levels [16]. The average election margin of victory across all Congressional districts in the U.S. is over 30% [17]. Future research should consider if university leadership is influenced by the political culture of their areas [18]. The lack of associations for religious universities is unexpected considering the common affiliation between religion and conservative political alignment.

Further research into university organizational decision dynamics is required to understand decisions in the context of politics and diversity. Universities with more racially diverse faculty were associated with earlier implementation of three NPIs. Organizational performance research of demographically diverse institutions shows that openness and diversity in beliefs were found to promote social integration and enhance organizational performance and decision-making [19]. Conservative university presidents network with community and business organizations more than their liberal colleagues [10]. These community relationships and local political ideology may affect university leadership NPI decisions given the negative economic impact of NPIs on adherence [2].

The impact of local public health orders on university NPI adoption should be explored further. Although state-level SOEs showed no statistical significance in this study, local SOE and SAH orders should be examined. From 2008 to 2017, over 56,000 local health department jobs were terminated; and public health budget cuts had a clear negative impact on COVID-19 NPI implementation [20]. Local public health orders were not available in the data set.

Disease transmission trajectories at universities and nearby small communities may be similar [21]. Universities in town and rural settings closed campus housing and moved employees to remote work more slowly. Delays in town and rural settings may increase risk of community spread to communities near university campuses. Universities with schools of public health and medical schools were not associated with decision timing. The role university public health and clinical resources should be examined to determine how they collaborated with university decision makers.

There are study limitations due to the data which used publicly available university information. Some missing university NPI decision data may represent lack of publicly available data rather than a universities’ inaction. It also may be that no evidence was found because an NPI decision was reached indirectly through a multi-university system policy, or government authority. Only universities with more than 5000 enrolled students were included due to limitations of the data set. The response and behavior of smaller universities, and those in small town and rural locations may be different. NPIs with variables that failed the survival analysis log-rank assumption test may be influenced by other factors such as timing of global, national, and local public health orders. Standard Cox proportional hazard models do not assess the outcomes where universities did not announce implementing an NPI during the study period but an alternative missing data approach was provided in the supplemental table. This results in the exclusion of NPI non-implementors from the analysis. However, a correlation analysis also found that there was no significant positive or negative correlation between the groups that did and did not implement the NPIs. Therefore, no adjustments were made for missing university decisions and dates.

## Conclusions

Universities generally followed public health and government guidance to implement targeted, layered application of multiple partially effective non-pharmaceutical interventions before explosive COVID-19 community spread. The timing of university NPI decisions varied by regional politics, faculty demographics, university governance, campus setting, and prevalence of foreign students. Religious affiliation and presence of university health infrastructure were not associated with timing.

## Supplementary Information

**Additional file 1: Supplemental Table S1.** Hazard ratios with missing dates set to end of observation period (3/31/2021).

## Data Availability

The datasets analyzed are available in the study, “COVID-19 observations and accompanying dataset of non-pharmaceutical interventions across U.S. universities, March 2020”, 10.1371/journal.pone.0240786. Copyright:© 2020 Cevasco et al., An open access article distributed under the terms of the Creative Commons Attribution License, which permits unrestricted use, distribution, and reproduction in any medium, provided the original author and source are credited.
