# Supplementary material for: Survival analysis of factors affecting the timing of COVID-19 non-pharmaceutical interventions by U.S. universities
Source: BMC Public Health. 2021 Nov 2;21:1985. doi: 10.1186/s12889-021-12035-6 (PMC8562371; doi:10.1186/s12889-021-12035-6)
Supplement: Supplementary file 1 — Additional file 1: Supplemental Table S1. Hazard ratios with missing dates set to end of observation period (3/31/2021). [file 12889_2021_12035_MOESM1_ESM.docx]

**Supplemental Table**

Supplemental Table S1: Hazard ratios with missing dates set to end of observation period (3/31/2021)

| **Survival Analysis Models with Explanatory Variables** | **Relative risk**  **(95% confidence interval)** | **P** |
| --- | --- | --- |
| ***Move Online*** |  |  |
| **Private / Public** | **0.79 (0.64:0.98)** | **0.031** |
| **Democrat/Republican Governor** | **0.80 (0.73:0.88)** | **<0.001** |
| **Democrat/Republican Congressional House** | **0.64 (0.52:0.78)** | **<0.001** |
| **More / Less Diverse Faculty** | **0.76 (0.60:0.95)** | **0.017** |
| More / Less Diverse Students | 1.09 (0.87:1.37) | 0.466 |
| No/ Yes Health infrastructure | 0.89 (0.72:1.09) | 0.257 |
| **Fewer/more foreign students** | **1.22 (1.02:1.46)** | **0.029** |
| City-Suburb / Town-Rural | 0.92 (0.72:1.18) | 0.517 |
| Enrollment <20k / >20k | 1.25 (0.99:1.57) | 0.059 |
| **State Case Prevalence, Quartile 1** |  |  |
| Quartile 2 | 0.82 (0.64:1.05) | 0.124 |
| **Quartile 3** | **0.39 (0.30:0.50)** | **<0.001** |
| **Quartile 4** | **0.53 (0.40:0.69)** | **<0.001** |
| ***Campus Housing*** |  |  |
| **Private / Public** | **0.51 (0.39:0.67)** | **<0.001** |
| Religious / Not Religious | 1.27 (0.91:1.78) | 0.161 |
| **Democrat/Republican Congressional House** | **0.71 (0.58:0.87)** | **<0.001** |
| No/ Yes Health infrastructure | 0.98 (0.81:1.19) | 0.861 |
| Fewer/more foreign students | 0.96 (0.79:1.15) | 0.638 |
| City-Suburb / Town-Rural | 0.80 (0.62:1.04) | 0.098 |
| **State Case Prevalence, Quartile 1** |  |  |
| **Quartile 2** | **0.53 (0.41:0.69)** | **<0.001** |
| **Quartile 3** | **0.21 (0.16:0.28)** | **<0.001** |
| **Quartile 4** | **0.11 (0.08:0.15)** | **<0.001** |
| ***Cancel Travel*** |  |  |
| Private / Public | 0.83 (0.67:1.03) | 0.087 |
| **Democrat/Republican Congressional House** | 0.61 (0.50:0.73) | **<0.001** |
| Fewer/more foreign students | 1.00 (0.83:1.20) | 0.994 |
| **State Case Prevalence, Quartile 1** |  |  |
| **Quartile 2** | **0.41 (0.32:0.53)** | **<0.001** |
| **Quartile 3** | **0.30 (0.23:0.39)** | **<0.001** |
| **Quartile 4** | **0.14 (0.10:0.18)** | **<0.001** |
| ***Campus Closed*** |  |  |
| Religious / Not Religious | 1.34 (1.01:1.78) | 0.043* |
| **Democrat/Republican Governor** | **0.78 (0.71:0.85)** | **<0.001** |
| **Democrat/Republican Congressional House** | **0.68 (0.55:0.83)** | **<0.001** |
| **More / Less Diverse Faculty** | **0.65 (0.53:0.80)** | **<0.001** |
| **State Case Prevalence, Quartile 1** |  |  |
| **Quartile 2** | **0.32 (0.25:0.42)** | **<0.001** |
| **Quartile 3** | **0.21 (0.16:0.27)** | **<0.001** |
| **Quartile 4** | **0.11 (0.08:0.15)** | **<0.001** |
| ***Remote Work*** |  |  |
| **Democrat/Republican Governor** | **0.68 (0.62:0.75)** | **<0.001** |
| **Democrat/Republican Congressional House** | **0.77 (0.63:0.94)** | **0.012** |
| **More / Less Diverse Faculty** | **0.69 (0.54:0.88)** | **0.003** |
| More / Less Diverse Students | 0.86 (0.67:1.10) | 0.228 |
| No/ Yes Health infrastructure | 0.90 (0.73:1.10) | 0.310 |
| **City-Suburb / Town-Rural** | **0.76 (0.58:0.98)** | **0.038** |
| **Enrollment <20k / >20k** | **1.32 (1.06:1.65)** | **0.014** |
| **State Case Prevalence, Quartile 1** |  |  |
| **Quartile 2** | **0.51 (0.40:0.66)** | **<0.001** |
| **Quartile 3** | **0.25 (0.19:0.33)** | **<0.001** |
| **Quartile 4** | **0.15 (0.12:0.21)** | **<0.001** |

**Campus closure, Religious / Not Religious was not statistically significant (p = 0.087) when adjusting for spring break as a time varying covariate*

*Statistically significant results in bold text*
